# Syncope After Transcatheter Aortic Valve Replacement: Two Faces of Abnormal Intraventricular Conduction

**DOI:** 10.19102/icrm.2020.110301

**Published:** 2020-03-15

**Authors:** Cyrus Kocherla, Chard Ward, Phillip A. Horwitz, Alexander Mazur

**Affiliations:** ^1^Division of Cardiovascular Medicine, University of Iowa Hospitals and Clinics, Iowa City, IA, USA

**Keywords:** Atrioventricular block, bundle branch reentry, conduction abnormalities, transcatheter aortic valve replacement, ventricular tachycardia

## Abstract

Conduction system damage is the most common complication of transcatheter aortic valve replacement (TAVR), which frequently requires placement of a permanent pacemaker. Bundle branch reentry (BBR) is a well-recognized mechanism of ventricular tachycardia (VT) in the setting of abnormal intraventricular conduction. We describe a case of a patient with post-TAVR intraventricular conduction abnormalities who presented with intermittent advanced atrioventricular block and BBR VT and discuss the potential risks, diagnosis, and management of BBR after TAVR.

## Case presentation

**Figure 1:**
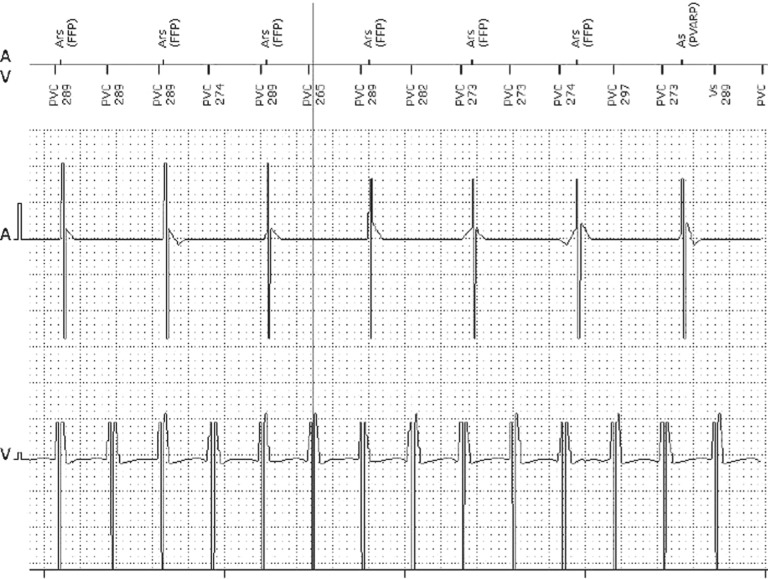
Stored pacemaker electrogram during syncope showing monomorphic tachycardia with VA dissociation. A and V: intracardiac recordings from the atrial and ventricular leads, respectively.

A 76-year-old female with a medical history of coronary artery disease and severe aortic stenosis who underwent transcatheter aortic valve replacement (TAVR) with a 23-mm Sapien S3 (Edwards LifeSciences, Irvine, CA, USA) prosthetic aortic valve presented to the clinic. Her preoperative electrocardiogram (ECG) showed no intraventricular conduction abnormalities (QRS duration: 90 ms) and a mildly prolonged P–R interval (212 ms). The postoperative period was complicated by the development of advanced conduction system abnormalities that manifested as alternating left and right bundle (RB) branch block (LBBB and RBBB, respectively) as well as transient complete heart block. Her postoperative echocardiogram showed normal left ventricular (LV) systolic function and a normally functioning aortic prosthetic valve. A dual-chamber permanent pacemaker was subsequently implanted. She presented three days later with complaints of recurrent brief episodes of dizziness and one episode of syncope. A review of the stored intracardiac electrograms from her pacemaker revealed multiple logged events of monomorphic tachycardia at average rates of 205 bpm to 223 bpm lasting from six seconds to 25 seconds. The timing of her syncopal episode corresponded with the longest logged tachycardia event. There was ventriculoatrial (VA) dissociation during these tachycardia events **(Figure 1)**, suggesting a ventricular tachycardia (VT) mechanism. Therefore, an electrophysiology study (EPS) was performed.

**Figure 2:**
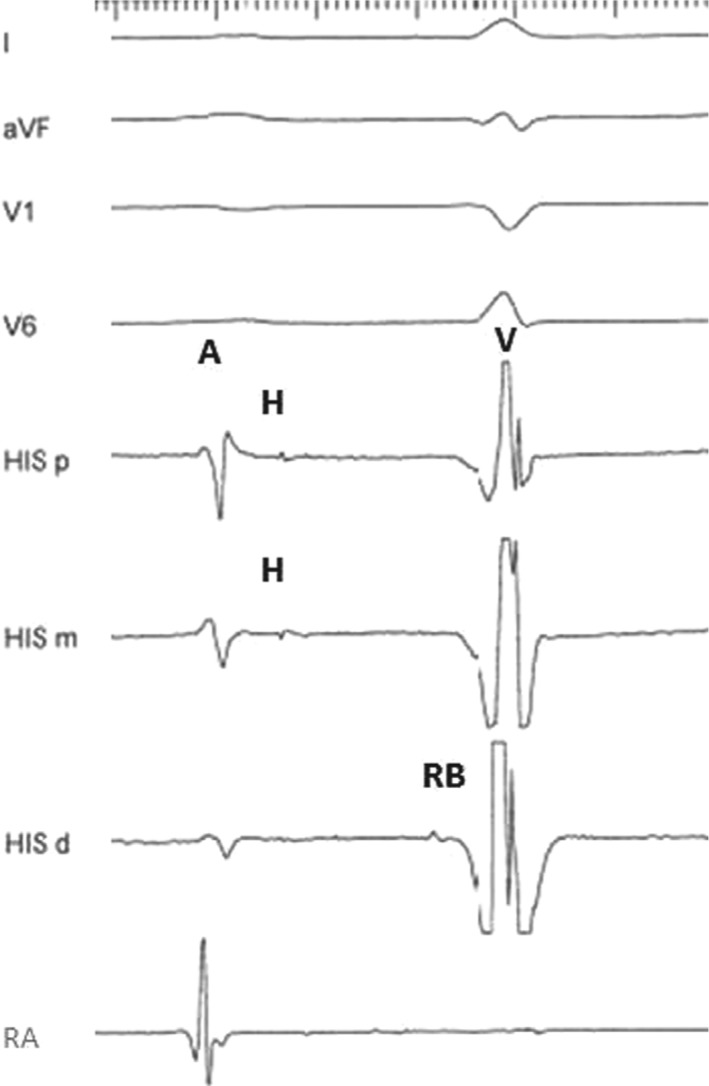
Surface ECG leads 1, aVF, V1, and V6 and intracardiac recordings from the His bundle and right ventricle during sinus rhythm. Note the markedly prolonged H–RB conduction time of 145 ms. A, His, RB, and V denote atrial, His bundle, right bundle, and ventricular electrograms, respectively.

**Figure 3:**
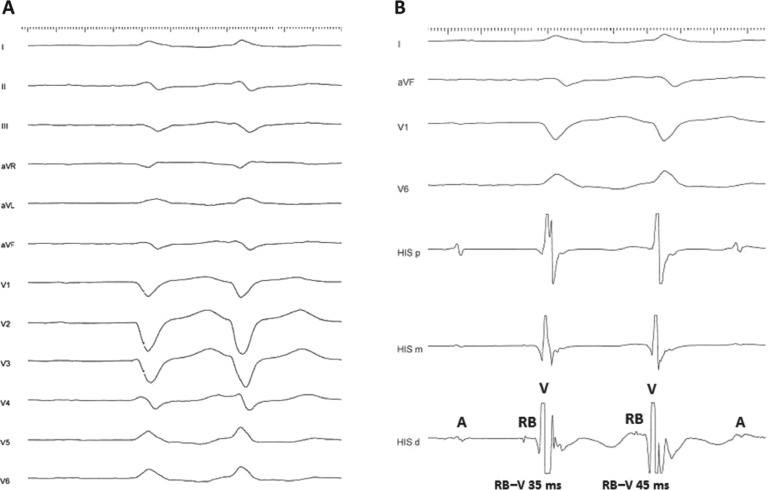
**A:** Twelve-lead ECG and **B:** corresponding intracardiac recordings from the His bundle and RV during spontaneous ventricular ectopy. Note the presence of LBBB QRS morphology similar to that in sinus rhythm and the RB potential preceding the QRS complex with an RB–V interval longer than that during sinus rhythm (45 versus 35 ms, respectively). A, RB, and V denote atrial, right bundle, and ventricular electrograms, respectively.

**Figure 4:**
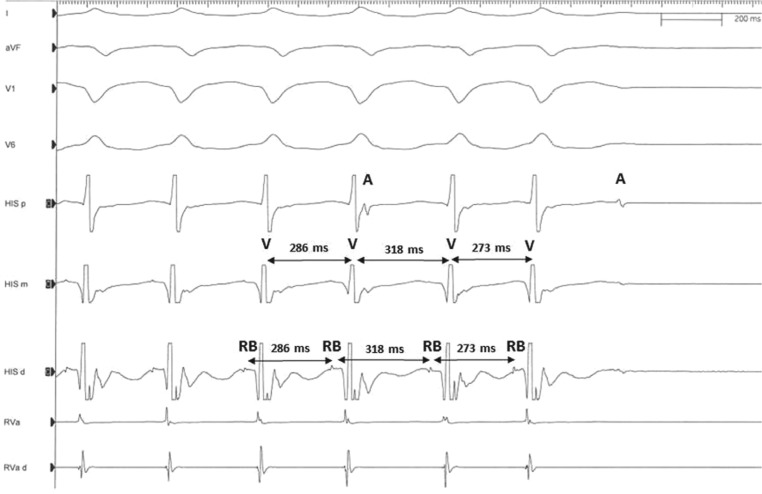
Surface ECG leads 1, aVF, V1, and V6 and intracardiac recordings from the His bundle and RV during induced tachycardia. The recordings are consistent with BBR VT. See text for discussion. A, RB, and V denote atrial, right bundle, and ventricular electrograms, respectively.

She presented to the electrophysiology laboratory in sinus rhythm with a cycle length of 950 ms, 1:1 atrioventricular (AV) conduction, and intermittent LBBB. For the EPS, her pacemaker was programmed to the VVI 45-bpm mode. The RB–ventricular (RB-V) interval was 35 ms. The proximal His-bundle potential could not be consistently obtained. A few recorded beats with the proximal His-bundle potential revealed a markedly prolonged intra-Hisian (H-RB) conduction time of 145 ms and an H–V interval of 180 ms **(Figure 2)**. The AV block cycle length was 420 ms. There was no VA conduction. Frequent spontaneous ventricular premature beats (VPBs) with the RB potential preceding V by 45 ms were noted. LBBB QRS morphology similar to that in sinus rhythm and an RB-V interval longer than that recorded in sinus rhythm strongly suggested a bundle branch reentry (BBR) mechanism of these VPBs **(Figure 3)**. A wide complex tachycardia identical to the above beats’ LBBB QRS morphology was induced with triple ventricular extrastimuli from the right ventricle **(Figure 4)**. The tachycardia was hemodynamically unstable and terminated spontaneously. Thus, pacing maneuvers during tachycardia were not feasible. The tachycardia mechanism was consistent with BBR VT based on the following electrophysiological findings: evidence of advanced intraventricular conduction defect during sinus rhythm; presence of VA dissociation during tachycardia; typical LBBB QRS morphology during tachycardia, which was similar to the patient’s LBBB QRS pattern in sinus rhythm; stable RB potential that preceded each ventricular activation with an RB-V interval during tachycardia longer than that recorded in sinus rhythm (35 ms versus 45 ms); spontaneous changes in the RB–RB intervals during tachycardia preceding similar changes in the V–V intervals; and spontaneous termination of the tachycardia with retrograde conduction block to the RB (V with no RB).

Following successful RB ablation (as evidenced by the development of RBBB), the index tachycardia was no longer inducible and there were no spontaneous VPBs with the RB potential preceding V. Also following ablation of the RB, an RBBB QRS pattern was noted. Additionally, 1:1 AV conduction with a P–R interval of 270 ms was observed. The RB potential could no longer be recorded at this point. No other sustained ventricular arrhythmia could be induced with programmed ventricular stimulation. The patient remained free from the presenting symptoms and arrhythmia over the ensuing three months.

## Discussion

We herein describe a case of a patient with post-TAVR intraventricular conduction abnormalities who presented with intermittent advanced AV block and BBR VT.

### Bundle branch reentry after corrective valve surgery and transcatheter aortic valve replacement

BBR is a well-recognized mechanism of VT in patients with advanced conduction system disease. Although structural heart abnormalities, particularly those resulting in cardiac dilatation, account for the majority of reported cases, BBR VT has been reported in patients without apparent structural heart disease or cardiac dilation.^1,2^ Although cardiac dilatation may potentially facilitate BBR by functional slowing of transseptal conduction between the bundle branches, a significant conduction delay within the His–Purkinje system is considered to be the major prerequisite for this arrhythmia mechanism. Aortic and mitral valve surgery are known causes of BBR VT. Because of the close anatomic proximity of the His bundle and proximal bundle branches to the valvular annuli, injury to them during corrective valve surgery is not uncommon. BBR has been reported in 10% to 29% of patients with inducible sustained VT after valve surgery.^3,4^ In this context, BBR VT usually manifests early in the postoperative period (typically within the first month after surgery) and commonly occurs in the setting of preserved LV systolic function. BBR VT has been recently reported in two cases after TAVR complicated by conduction abnormalities.^5,6^ Similar to in our case, both patients presented with arrhythmia soon after the procedure and had relatively preserved LV systolic function.

### Conduction system abnormalities after transcatheter aortic valve replacement

TAVR has become a commonly performed procedure for the treatment of severe aortic stenosis as an alternative to surgical aortic valve replacement in high- and moderate-risk surgical candidates. Recent data support broadened indications for this technology; thus, it is likely to emerge as a primary approach for severe aortic stenosis even in patients at lower surgical risk.^7,8^ Conduction abnormalities such as high-grade AV block and new LBBB are common after TAVR (pacemaker: 2%–51%; new LBBB: 4%–65%), and incidence rates have not decreased despite improvements in operator experience and the introduction of newer-generation valves in more recent years.^9,10^ An autopsy report has suggested necrosis of the His bundle and proximal LBBB due to mechanical compression by the expanded prosthetic valve to be the underlying pathology of post-TAVR conduction abnormalities.^11^ Reported rates of this complication have been higher with the use of self-expandable valves relative to those with balloon-expandable valves.^9,10^

### Post–transcatheter aortic valve replacement conduction abnormalities and the risk of sudden death and ventricular arrhythmias

The available data on the risk of sudden cardiac death (SCD) following TAVR are scarce. Although AV block is considered to be the major potential mechanism of SCD in this setting, cases of SCD attributable to ventricular arrhythmias have been reported.^12^ Most published TAVR series do not detail the incidence of SCD separately from that of cardiovascular mortality. One of the possible explanations is that the mode of death is frequently difficult to ascertain, particularly when performing a retrospective data analysis. In a recent analysis of prospectively collected data from 3,726 consecutive TAVR patients in 18 centers worldwide, the cumulative rates of SCD at one and two years of follow-up were 1.0% (95% confidence interval: 0.6%–1.4%) and 1.8% (95% confidence interval: 1.2%–2.4%, respectively. SCD accounted for 5.6% of total deaths and 16.9% of cardiac-related deaths in this patient population. An LV ejection fraction of 40% or less prior to TAVR (hazard ratio: 1.93; 95% confidence interval: 1.05–3.55; p = 0.033) and the development of new LBBB (hazard ratio: 2.26; 95% confidence interval: 1.23–4.14; p = 0.009) were independent predictors of SCD.^13^ Further, a QRS duration of more than 160 ms was found to be the best discriminator for predicting SCD in post-TAVR LBBB patients (area under the curve: 0.64; standard error: 0.09). In this cohort, one of 10 patients died suddenly within one year after TAVR (rate of SCD of 9.9% at one year of follow-up). Although the exact mechanism of SCD (ie, progression to AV block, ventricular arrhythmia, or other) could not be determined, the presence of a permanent pacemaker appeared to have no impact on SCD risk in this analysis. In the prospective Ambulatory Electrocardiographic Monitoring for the Detection of High-degree AV Block in Patients with New-onset Persistent LBBB after TAVI (MARE) study, 103 patients with new-onset persistent post-TAVR LBBB were implanted with an insertable loop monitor and were followed up with for one year.^14^ Progression to high-degree AV block was noted in 15 (15%) patients, while 10 (10%) received a pacemaker for symptomatic bradycardia. VT was documented in 13 (13%) patients including 12 cases of nonsustained (duration: 6–11 seconds) and one case of sustained VT. Based on these findings, two patients received implantable cardioverter-defibrillators (ICDs) and four patients were treated with antiarrhythmic medications.

### Post–transcatheter aortic valve replacement conduction abnormalities and the risk of bundle branch reentry

The incidence of BBR VT after TAVR is unknown. This issue might be compounded by the fact that EPS is currently not routinely performed in many patients with documented VT prior to ICD placement, particularly in the setting of reduced LV systolic function. Given the frequency of conduction system complications following TAVR, it is conceivable to speculate that some cases of BBR remain undiagnosed. Unrecognized BBR VT may result in sudden death or cause significant morbidity secondary to recurrent syncope or ICD shocks. Antiarrhythmic medications are usually ineffective, whereas the ablation of a bundle branch is a highly successful and potentially curative procedure.^15^ There are currently no practical approaches established for identifying patients at risk for BBR VT after TAVR. The utility of EPS in patients with post-TAVR new-onset intraventricular conduction defects for evaluating the risk of ventricular arrhythmias (including BBR) or progression to high-degree AV block is yet to be determined.

### Role of implantable cardioverter-defibrillators in patients with bundle branch reentry

The available data from small retrospective series suggest that the long-term outcome of patients with BBR VT treated by catheter ablation is primarily driven by underlying cardiac pathology, and the decision about ICD placement should be guided by conventional risk markers for sudden death such as LV function or evidence of scar-related ventricular arrhythmias (spontaneous or inducible), among others.^3,16–19^ The role of ICDs in BBR patients without these risk markers is less clear. Limited evidence indicates that these patients may have a favorable prognosis following the successful ablation of BBR.^1,2,19^ Our patient had preserved LV systolic function and no inducible VTs other than BBR. Thus, we elected not to pursue upgrading her pacemaker to an ICD.

In summary, our case indicates that BBR should be strongly considered as a potential mechanism of syncope or documented VT, particularly occurring in the early postoperative period, in patients after TAVR procedure complicated by new intraventricular conduction defects.
